# Addressing the Problem of Brain Death Misdiagnosis

**DOI:** 10.1017/jme.2025.10107

**Published:** 2025

**Authors:** Marjorie Fitzsimmons, Katherine Drabiak, Prithvi Shetty

**Affiliations:** 1 USF Health Morsani College of Medicine, Tampa, United States; 2 College of Public Health, University of South Florida Health, Tampa, United States

**Keywords:** brain death, death by neurological criteria, Uniform Determination of Death Act, Brain Death* / legislation & jurisprudence, Brain Death* / diagnosis, ethics, clinical

## Abstract

Recent literature describes the controversy relating to brain death/death by neurological criteria (DNC), which some have referred to as “widely accepted, but not universally supported.” This article provides an overview of differences in state laws relating to DNC and describes recent proposals to reform the definition of brain death. In 2023, the American Academy of Neurology (AAN) issued clinical guidelines stating that clinicians may declare a patient DNC despite evidence of neuroendocrine function — a position that directly conflicts with state law requirements for determining death. This article offers a critical analysis of AAN guidelines, an update on proposals to reform the Uniform Determination of Death Act, and explains why policy discussions should include how DNC exams occur in practice. Research suggests there are flaws with current clinical testing methods, which contributes to two separate problems: (1) false positives from insufficient testing, and (2) inadvertent misdiagnosis from unintentional errors. Together, this has produced confusion and reduced public trust in the concept of brain death. This article provides recommendations to clarify and retain the current legal standard for brain death, explains the ethical importance of accurate standards for determining DNC, and offers practical solutions to reduce errors.

## Introduction

Since 1968, the Harvard Criteria formed the basis for determining brain death/death by neurological criteria (DNC) in cases where patients experienced severe and irreversible brain damage despite cardiorespiratory function with assistance of life-sustaining interventions.^1^ The Uniform Determination of Death Act (UDDA) provides a clear and standardized definition of death, and all fifty states have adopted a form of the UDDA.^2^ While current laws are generally modeled after the UDDA, significant differences still exist between states.

Recent literature describes the controversy related to determination of DNC, which some have referred to as “widely accepted, but not universally supported.”^3^ Drost et al. estimate approximately 2% of all in-hospital deaths constitute death by neurological criteria, and sound public policy requires a bright line when determining whether a patient is alive or dead.^4^ Physicians’ legal duties differ for a patient who is alive — such as obtaining informed consent for interventions or removing support — while duties to patients who are deceased relate to custody of a body, or organ donation.^5^ At the societal level, clear demarcation of death indicates whether a person has constitutional rights, and corresponds to a variety of specific interests relating to execution of wills, marriage status, insurance payments, and inheritance.^6^ The problem of nonuniformity where a patient could be dead in one state and alive in another state reduces public confidence and trust in the medical profession.^7^

This article provides an overview of differences between state laws, describes key points of American Academy of Neurology’s (AAN) clinical guidelines, and explains the mismatch between AAN’s guidelines and what is legally required to diagnose death.^8^ Current standards for DNC testing are insufficient to measure irreversible cessation of all functioning of the entire brain, which can lead to false positives.^9^ Compounding this problem, research suggests that physicians who conduct DNC exams skip or misinterpret required clinical components, which contributes to inadvertent misdiagnosis and erroneous declarations of DNC.^10^ This article recommends retaining — and clarifying — what is legally required for determining DNC, describes methods to reduce potential errors, and explains why the current legal standard upholds the ethical practice of medicine.

## Determining Brain Death/Death by Neurological Criteria

### Clinical Standards for Brain Death/DNC

The UDDA defines death as irreversible cardiopulmonary cessation or “irreversible cessation of all functions of the entire brain, including the brain stem” and states this determination must be made in accordance with “accepted medical standards.”^11^ State laws modeled after the UDDA are designed to provide an objective and consistent standard that measures the loss of *all brain function* required for integrated functioning of the human body.^12^ Clinical standards expand upon the legal definition, provide guidance for physicians, and assess whether the patient meets the established legal criteria for death.

#### American Academy of Neurology Clinical Guidelines

AAN current guidelines for determining brain death provide physicians with a standard evaluation protocol, which some neurologists assert should constitute the reference to “accepted medical standards.”^13^ The guidelines establish clinical criteria for determining cessation of brain function, which requires the absence of brain stem reflexes, presence of a catastrophic neurological injury, and absence of respiratory drive tested with a CO_2_ challenge.

AAN recommends a multiple step process using the patient’s history, laboratory tests, imaging, and a clinical evaluation to determine the cause of the neurological injury.^14^ Then, physicians must rule out mimicking medical conditions such as the presence of central nervous system (CNS)-depressant drugs; use of neuromuscular blocking agents; severe electrolyte, acid-base, or endocrine disturbances; and rule out the possibility of hypothermia. After these prerequisites are accomplished, the guidelines set forth steps for neurological examination to assess responsiveness to pain and patient reflexes. For adults, AAN states that if a sufficient period has passed to exclude the possibility of the patient recovering, then one neurological exam is sufficient.^15^ This exam should be followed by an apnea test, which constitutes a critical component to evaluate the integrity of the medullary respiratory center’s response to rising blood concentration of CO_2_.^16^ If the results of the brain death evaluation are inconclusive or the apnea test cannot be performed, AAN recognizes that ancillary tests such as an EEG, cerebral angiogram, or nuclear scan may be used.^17^ However, physicians should be aware of the potential for false positives when ancillary tests are used for brain death diagnosis, and that these tests are not necessary to diagnose brain death and should not replace a standard neurologic examination.^18^

#### Controversy in AAN’s Clinical Guidelines

In 2023, AAN updated its clinical guidelines with several notable modifications.^19^ First, AAN erased the term “irreversible” cessation of all neurological function and replaced it with the term “permanent,” to indicate the patient lost neurological function and physicians will not use medical interventions to attempt to restore function.^20^ Next, AAN reaffirmed and strengthened its stance that physicians do not need to obtain informed consent (unless stipulated by specific state laws or hospital policies) to conduct the DNC exam and apnea testing.^21^ The clinical guidelines also addressed cases of pregnant patients, stating that pregnancy itself is not a contraindication to DNC. Depending on prior wishes of the patient and surrogate, the surrogate may choose to continue or discontinue supportive measures.^22^ Finally, AAN reaffirmed its position that physicians may declare a patient DNC despite persistence of neuroendocrine function.^23^

The problem with several components of these guidelines is that they patently conflict — and attempt to override — the legal definition of death and legal requirements pertaining to informed consent in states that are otherwise silent on the narrow issue.^24^

Physicians and ethicists who favor current clinical standards set forth by AAN declare that it constitutes a reliable tool. Until 2023, AAN asserted there had been no cases in which neurological function has been recovered in a patient after being declared brain dead using the criteria.^25^ Nair-Collins and Joffe — critics of current AAN guidelines — point out medical literature repeats the common refrain, declaring that if physicians follow clinical standards this prevents false diagnosis, false positives do not occur, and the risk of mistake is “infinitesimal.”^26^ Some ethicists point out this entails a self-fulfilling prophecy, because physicians remove supportive interventions following declaration of DNC.^27^ In 2023, AAN conceded two cases of false positive misdiagnosis.^28^

### Legal Standards for Brain Death

All states have adopted laws that recognize DNC, but significant variation still exist among states.^29^ Key differences include issues such as: who may perform the DNC exam; whether the physician must obtain informed consent from a surrogate to perform a DNC evaluation; how to address accommodations or requests for exemption from DNC from surrogates; and how to manage pregnant patients who are DNC.

#### Specifying Protocol and Provider

One variation among state laws is whether the law specifies certain protocols for DNC, such as procedures for administering tests, the number of physicians required to assess the patient, and the qualifications of these physicians.^30^ For example, currently only Florida, New Jersey, and Virginia require a physician in neurology or critical care medicine to make the declaration of brain death.^31^ Other states such as New York specify that a physician is not required to be a neurologist or consult with a neurologist before determining brain death.^32^ Research suggests that physician specialty varies how the physician conducts the exam and impacts adherence to clinical guidelines.^33^

#### Informed Consent for DNC Evaluation and Apnea Testing

In the vast majority of instances, physicians must obtain informed consent to perform an evaluation, examination, or intervention on any patient. Some physicians and ethicists assert that this general principle should not apply to DNC examinations, or should constitute an exception.^34^ Other experts maintain informed consent is legally and ethically required for all patient exams and interventions, including the determination of DNC.^35^

Only a few states have addressed the issue of whether informed consent is required from the patient’s surrogate to perform the brain death evaluation including apnea testing. Pope has described how litigation relating this issue has resulted in opposite outcomes.^36^ Courts in Virginia and Nevada held that consent was not necessary since physicians have an interest in determining whether a patient is alive or dead.^37^ Court cases in Montana and Kansas, however, held that consent for apnea testing was legally required, noting that physicians must obtain informed consent to perform medical procedures on patients.^38^ In Nevada, the state legislature amended the law following *In re Guardianship of Hailu* as a mechanism to avoid further conflicts over the question of informed consent and what constitutes “accepted medical standards.”^39^ Nevada specifies that the law incorporates AAN guidelines as the currently accepted medical standards, which state that informed consent is not required to conduct apnea testing.^40^ New York addresses the issue in guidance, stating that physicians do not need to obtain consent to determine brain death.^41^ The vast majority of states, however, have not addressed this issue.^42^

Many physicians and AAN assert that physicians have the responsibility to perform a brain death evaluation without obligation to obtain informed consent for any component of the test including apnea testing.^43^ Pope maintains that informed consent is not required from a surrogate to perform a DNC evaluation and this constitutes settled law and practice.^44^ AAN’s guidelines for determining brain death specify that testing for apnea is essential to diagnosing brain death and this can only be assessed reliably by disconnecting the ventilator.^45^ Requiring consent for DNC testing would make it impossible for physicians to diagnose brain death and assess patient status as alive or dead.^46^

Some physicians and legal scholars assert DNC tests constitute patient assessment or evaluation, and should be distinguished from providing healthcare.^47^ Similarly, AAN reasons that if consent is not required for assessing cardiopulmonary death, it should also not be required to evaluate neurological death.^48^ Alternatively, Pope asserts that determining the fundamental status of whether a patient is alive or dead should fall within a legal exception to obtaining informed consent, comparable to a legal exception that permits physicians to treat patients in an emergency without informed consent.^49^ While DNC tests including the apnea test do not provide medical benefit, they do answer the important question of whether a patient is alive or dead.^50^ Finally, physicians maintain that apnea tests are safe when properly performed and constitute the final confirmatory step for patients that already appear to fit the criteria for DNC.^51^

Similarly, Lewis et al. assert that physicians have a fundamental responsibility to perform DNC evaluations unilaterally without obtaining surrogate consent.^52^ Instead, they suggest that physicians may keep the patient’s family informed of patient’s status.^53^

Despite AAN declarations, other physicians and ethicists assert that informed consent from a surrogate is legally and ethically required not only for apnea testing, but the entire DNC evaluation.^54^ Although physicians may indeed be performing DNC exams without informed consent, Paquette et al. argue this in fact violates accepted medical standards.^55^ Conducting clinical exams, interventions, or tests without informed consent can constitute both malpractice and battery.^56^ Any medical test or evaluation requires informed consent, which permits the patient or surrogate to assess the benefits and risks of the intervention. The emergency exception in the law permits treating patients without consent who would be harmed by lack of immediate intervention. Truog and Tasker assert this is disanalogous: rather than saving the patient, the apnea test could cause the patient to suffer harm.^57^ Truog and Tasker note that DNC evaluations would not fall under the category of a general consent to treatment, which encompasses using a general informed consent for related interventions that benefit the patient.^58^

Elaborating on the concept of harm, physicians and ethicists describe distinct and significant risks and complications from apnea testing, noting that the physiological changes from the test may run counter to therapeutic objectives for managing a patient in a deep coma, worsen intracranial pressure and neurological injury, result in myocardial infarction, and increase risk of iatrogenic harm.^59^ Apnea testing constitutes a non-emergent, non-beneficial intervention, and may constitute final *coup de grâce*: it can induce patient death in patients who are suffering from severe neurological injuries, but are still alive.^60^

Several experts acknowledge that physicians are reluctant to yield authority by requiring informed consent to perform DNC evaluations, or argue that most do not obtain consent in practice.^61^ However, AAN recognizes that a patient is legally alive at the beginning of a DNC exam.^62^ This admission is significant, and a reminder that patients who are alive have distinct legal rights to accept or refuse medical interventions and examinations that are exercised by a surrogate providing (or declining) informed consent. Even if forgoing consent is common practice among physicians or obtaining consent is difficult, this does not make it legally permissible to override this requirement.

Physicians who try to follow AAN’s clinical guidelines face tension and confusion because AAN’s recommendations conflict with the legal requirement for informed consent. Informed consent serves as a mechanism to guard against physician paternalism, respect patient autonomy, the right to bodily integrity, the moral status of each person, and uphold trust in the medical profession.^63^ To facilitate determining patient status while upholding the principle of informed consent, Berkowitz and Garrett propose an informed dissent, or opt-out system.^64^ In other contexts, however, McKay and Robinson suggest that opt-out systems or even nudges using active choice models that steer individuals to choose a specific outcome undermine autonomy and are morally problematic.^65^

#### Accommodations and Exemptions

State laws vary relating to whether they allow for accommodations when a patient’s family member or surrogate disagrees with using neurological criteria to declare death for moral or religious reasons. Some institutions may provide short-term supportive measures following a DNC evaluation to allow families to gather for the grieving process. California and New York law requires physicians to provide families with a period of accommodation if there are religious or moral objections to determination of DNC, but the laws specify that these accommodations should be for a reasonable period of time and the accommodation does not change the status of the patient as legally dead.^66^ Nevada law, on the other hand, mandates that life-sustaining treatments should be withdrawn within 24 hours after brain death is declared and specifies that the families may be responsible for any costs associated with supportive measures.^67^ New Jersey is the only state that recognizes an exemption from declaring death on the basis of neurological criteria if doing so would conflict with the religious beliefs of the patient.^68^ This exception in New Jersey combined with litigation over the determination of death by DNC perpetuates confusion over the medical and legal status of patients who are DNC.^69^

AAN asserts that autonomy is not absolute, and patients do not have the right to receive desired but unjustified medical treatment, including life-sustaining treatment.^70^ AAN maintains that death is a biological reality that physicians can determine objectively by uniform standards.^71^ Accordingly, Magnus et al. portray objections to DNC as familial refusal to accept a biological reality and reliance on misinformation.^72^ Physicians also have no ethical obligation to provide treatment to a deceased person, and AAN maintains that providing indefinite accommodations may even cause unnecessary harm defined as mistreatment of the newly deceased’s body. In cases of true brain death, this may also perpetuate false hope, prolong the grieving process, and deprive the decedent of dignity.^73^ Some physicians assert that eliminating exemptions will enhance clarity, prevent inconsistencies, and uphold physicians’ obligation to provide a timely and accurate record of death.^74^

On the other hand, some ethicists and physicians support a legal exemption from DNC. In some instances, families object to DNC on the basis of the patient’s religious beliefs. Yanke et al. note that while the belief that life does not end until cardiopulmonary death is not widespread, it is present in multiple religious traditions such as Orthodox Judaism, Buddhism, Islam, Native American religions, Shintoism, and some Christian beliefs.^75^ Shewmon asserts surrogates may object not only on religious grounds, but on philosophical grounds arising from recognition that neurological death does not equate to biological death.^76^ These tensions can lead to conflicts, and raise additional scrutiny about how to reconcile organ donation and from patients who are DNC and whether this warrants reassessing the dead donor rule.^77^

Court cases related to families objecting to brain death are typically inefficient and costly, but the frequency of surrogates seeking an exemption is rare.^78^ Some ethicists propose that state laws permitting an exemption similar to New Jersey would prevent these conflicts from arising, preserve patients’ rights to make decisions regarding their own healthcare, and decrease public mistrust.^79^ Notably, recognizing an exemption does not translate to all surrogates choosing extended supportive measures; this may result in surrogates making a decision to withdraw supportive measures to allow death by cardiopulmonary criteria.^80^ Finally, some objections to DNC may be reduced by addressing the problems of false positives and inadvertent misdiagnosis.

#### DNC and Pregnant Patients

Biel and Durrant note that states also differ relating to treatment of pregnant patients who fit the criteria for DNC when determining whether to maintain the patient on support to gestate the pregnancy or terminate support. Minnesota and Oklahoma specify physicians should follow the wishes of the pregnant patient or surrogate.^81^ Nevada state law, on the other hand, requires maintaining support for a pregnant patient if the physician determines it is probable that the fetus will develop to a live birth.^82^ Many states have laws pertaining to life support for pregnant patients, but it is unclear how this would apply to pregnant patients who are DNC.^83^ In Texas, *Munoz v. JPS Hospital* held that the law stating life-sustaining treatments should not be withdrawn from pregnant patients excluded patients who were DNC, because treatment cannot be considered “life-sustaining” if the patient is already dead.^84^ Despite the complexity of this issue, more than 90% of hospitals in the United States offer no guidance about fetal management after maternal DNC in their policies.^85^

## Problems with Current Brain Death Standards and Proposals for Revision

### DNC/Brain Death Criteria and False Positives

#### DNC Exams Do Not Measure Irreversible Loss of Function of the Entire Brain

Physicians, ethicists, and legal scholars each recognize that the controversy stems from the fact that prevailing medical standards do no not measure what the law requires and there is currently a lack of alignment between medical assessment and legal requirements.^86^ Sulmasy et al. suggest that three categories of cases challenge the concept of DNC: patients who are pregnant who continue to gestate a fetus; patients experiencing “chronic brain death” whose bodies persist for months following DNC diagnosis; and high profile cases like Jahi McMath.^87^ In the case of McMath, for example, Pope contends that physicians performed the DNC examination in accordance with appropriate clinical standards, but that clinical guidelines were insufficient to measure irreversible cessation of all functions of the entire brain.^88^ Instead, the current clinical standards are only testing for *partial* brain death, and infer the loss of function of the rest of the brain.^89^ Similarly, some argue that current guidelines are not designed to assess the irreversibility and totality of the neurological injury, which means physicians may declare patients DNC who do not meet the legal definition.^90^

More precisely, current clinical guidelines for the DNC exam do not assess hypothalamic function.^91^ Nair-Collins highlights that patients who fit the clinical criteria for DNC may still have several categories of preserved neurological functioning in regions such as the hypothalamus (osmoregulation), endocrine function (pituitary functioning), and cortical electrical activity.^92^ Any function of these areas of the brain conflicts with the legal definition of DNC.^93^ Importantly, if patients are maintained on ventilation, this support enables their bodies to continue biological processes such as cellular respiration, growth, waste excretion, pregnancy gestation, wound healing, response to infection, and endocrine response to incisions.^94^ Nair-Collins and Joffe suggest that preserved neurological function could be measured by proxy, by examining whether the patient has experienced medical conditions that would normally be regulated by the hypothalamus (e.g. diabetes insipidus) or the pituitary gland (e.g. thyroid deficiency or adrenal failure).^95^ Estimates for cases in which patients retain hypothalamic function vary, ranging from estimates of 9% to up to 90% of patients who were declared DNC.^96^

This means even if physicians correctly follow clinical guidelines, this will result in a significant percentage of patients who are declared DNC who have not experienced total and irreversible loss of all function of their entire brain. In plain terms, current diagnostic methods yield false positive results — physicians declare patients dead who are not in fact dead.

In these cases, it is more accurate to diagnose that the patient suffered a profound brain injury rather than total loss of all neurological functioning. There is a delicate line between patients who are nearly dead versus patients who are actually dead. Miller et al. assert although medical interventions can assist respiration, circulation, and other bodily functions, these processes could not be maintained on a corpse and conclude that the patient is still biologically alive.^97^ Despite this, many point out that an accurate DNC diagnosis does not require that all neurological cells are destroyed or nonfunctional.^98^ Rather, Sulmasy et al. suggest DNC occurs when the locus of control and integration is no longer the patient, but the clinicians tending to the patient, where the patient has lost the capacity to initiate and maintain bodily functions.^99^

#### AAN’s Response to Neuroendocrine Functioning

In 2019, AAN updated its guidelines to recognize this dilemma, stating that neuroendocrine functioning may persist in patients who otherwise meet the clinical criteria for DNC.^100^ AAN stated clinical standards *should still classify these patients as dead (DNC) despite not meeting the present legal definition.* ^101^ AAN reasons that since the patient is beyond recovery or *near death*, the law should permit an exception.^102^ It affirmed this stance again in 2023, but omitted the statement that this guideline does not meet the legal definition.^103^

AAN states that the demise of organ systems and the body is inevitable without supportive measures to maintain perfusion and ventilation.^104^ Some physicians maintain that dying is a process, the brain holds a significant role in mediating bodily organs and systems as a whole, and purification will necessarily follow the diagnosis of DNC.^105^ Other physicians may justify AAN’s position by dismissing the role of the hypothalamus or attempting to deny it constitutes part of the brain.^106^

#### The Problem with AAN’s Clinical Guidelines

AAN guidelines are in clear conflict with the legal definition required for determining death. As Sulmasy et al. point out, statements that deny the location or critical function of the hypothalamus are physiologically and anatomically false.^107^ This also closely relates to AAN’s revision of declaring patients DNC based on “permanent” rather than “irreversible” cessation, which indicates the physician’s decision to *forgo* certain testing to determine whether the patient’s condition is irreversible.^108^ This is distinct from using clinical criteria to assess whether the patient’s condition *could* be reversed. AAN’s clinical guidelines reflect expanding the category of “dead” to include patients who are *close* to death, but under the law are still alive. AAN’s guidelines purport to unilaterally erase two key requirements in both the UDDA and state laws: the requirement that the patient has suffered a loss of *all* neurological functioning of the entire brain, and the requirement that this loss is *irreversible.*

Clinical guidelines are designed as a blueprint for consistency and best practices, but do not have the authority to rewrite or override the current law. This creates immense confusion and an untenable situation: if physicians abide by AAN’s guidelines and declare patients DNC who still have neuroendocrine functioning, this not only constitutes misdiagnosis, but violates state laws.

Experts agree that ideal clinical standards for determining DNC must be consistent, reliable, and accurate. The appropriate response, then, should be to improve the testing methods rather than trying to revise the definition of death.^109^ American College of Physicians stated it concisely: clinical tests do not define death, but rather confirm whether it occurred.^110^

### Policy Proposals to Revise the UDDA

To address the disconnect between law and clinical practice, multiple stakeholders submitted proposals to the Uniform Law Commission, which convened to consider potential revisions to the UDDA in 2023.^111^ The goal of revising the model law was to provide an updated template that states could choose to adopt, which would modify how state law defines brain death/DNC.

Lewis et al. proposed a revised UDDA that would change the model law definition to permit physicians to declare death despite neuroendocrine functioning, mirroring AAN guidelines.^112^ Similarly, Omelianchuk et al. recommended revising the UDDA to classify DNC in a more expansive manner, defined as brain injury leading to permanent loss of the capacity for consciousness, the ability to breathe spontaneously, and lack of brainstem reflexes.^113^

Other experts have suggested a range of solutions aimed at acknowledging that legal and clinical criteria are not aligned.^114^

Some physicians proposed abandoning the concept of brain death. A form of this option would clarify that the DNC exam assesses only neurological functioning, but that this does not equate to biological death.^115^ Some of these concerns, however, may be ameliorated by more precise testing to eliminate false positive diagnoses.

Previously, legal scholars have recommended recognizing that DNC more appropriately constitutes a legal fiction serving a bright line to determine death, and argued that the law should permit certain exceptions.^116^

However, Sulmasy et al. note permitting exceptions or opt-outs may cause confusion or greater inconsistency.^117^ This also avoids addressing the critical shortcomings in the current testing methods and working to improve clinical guidelines. Instead, American College of Physicians suggested retaining the current legal definition of brain death, but improving DNC exam standards.^118^

The Uniform Law Commission held discussions and considered proposals to modify the UDDA from 2022-–2023. Following discussion, the Uniform Law Commission drafted a proposed revised UDDA that would have enshrined AAN’s guidelines as the new model law. Multiple physicians, ethicists, and legal scholars submitted comments to the proposed revision, with a variety of objections.^119^ As of this writing, the Uniform Law Commission indefinitely paused any revisions to the UDDA based on the inability to reach a consensus.^120^

It is worth noting that AAN published updated clinical guidelines at the end of 2023, shortly after the Uniform Law Commission declined to modify the UDDA.

### The Remaining Problem of Inadvertent Misdiagnosis

There is a significant body of literature that discusses the problem of false positive diagnoses of DNC, despite physicians following standard steps for DNC exams according to AAN standards.^121^ However, research also suggests another compound problem of inadvertent misdiagnosis, defined as physicians omitting key steps of the DNC exam or misinterpreting what test results signify.^122^ Despite insufficiency of AAN guidelines, skipping critical steps worsens the potential for errors. Bernat and Brust assert these practices are not innocuous but can produce serious consequences and result in misdiagnosis.^123^

Some variation is expected with how physicians perform DNC exams, part of which may be attributed to different protocols across institutions. Some institutions mandate ancillary tests for all patients, recommend tests that are not endorsed by AAN, or alternatively do not specify when ancillary testing should be used.^124^ Other variations include institutional policies relating to who can diagnose brain death, such as specifying the attending physician or allowing diagnosis by an advanced practice provider.^125^

However, some differences in how physicians perform DNC exams in adults are more than mere variation, but suggest physicians are omitting necessary steps or misinterpreting key results. This includes missing or skipping steps such as: establishing the absence of hypothermia or drug intoxication, performing an apnea test, or repeating the examination before declaring brain death.^126^

In an education simulation for residents in critical care and neurology, Hocker et al. found only 39% of participants considered potential confounders such as presence of drugs, alcohol, or CNS depressants, and only 58.5% considered whether further treatment could benefit the patient before initiating the DNC exam.^127^ During the DNC exam, only 22% of participants checked for spontaneous respiration before initiating the apnea test, and during the apnea test only 43.8% of participants recognized the simulation mannequin breathing.^128^ These findings suggest physicians may proceed to a DNC exam in cases where it may not be indicated and the patient suffers from a mimicking condition, or may not recognize when a patient demonstrates respiration and is still alive. Errors such as not considering potential confounders or waiting an insufficient amount of time before brain swelling reduces could have serious implications when the patient who is declared DNC is also part of the organ donation process, because the patient may begin undergoing organ retrieval while still alive. Although a theoretical concern, rare cases allege that patients were erroneously declared DNC and scheduled for organ retrieval, but did not meet the legal criteria for death.^129^

Braksick et al. surveyed physicians practicing at academic medical centers who completed DNC exams and found similar significant deviation from clinical guidelines.^130^ Braksick et al. found 85.3% reported self-competence in completing a brain death exam, and 76.1% of physicians received training on brain death examination.^131^ However, only 25% of participants reported conducting the DNC exam according to AAN guidelines.^132^ While the majority of participants completed the apnea test, 10.4% of participants did not.^133^ Braksick notes that the apnea test constitutes a critical component of the DNC exam unless contraindicated by patient condition, and omitting the apnea test results in an incorrect determination.^134^ Participants also relied on ancillary tests in 30.3% of evaluations as a mechanism of certainty, to avoid liability, or belief such tests were required by the institution’s policy.^135^ Problematically, 28.3% of participants reported ordering ancillary testing to confirm death when a patient breathes during the apnea test — *which is an indication the patient is alive* — and cannot be declared DNC.^136^

Braksick et al. conclude that the results suggest not merely practice variation, but that physicians may be incorrectly performing DNC exams leading to erroneous results.^137^ Moreover, Braksick et al. note the significance of these findings from physicians practicing at an academic medical center, which suggests the possibility of perpetuating inaccurate training for medical students, residents, and fellows.^138^

Chen and LaBuzetta demonstrated that uncertainty and lack of knowledge also impacts the rest of the care team, such as nurses and medical students.^139^ Chen and LaBuzetta found 84% of nurses and about 33% of medical students participated in family discussions, observations, and assisted with components of the DNC exam, but reported gaps in their understanding of DNC, which impeded accurate, clear communication with family members.^140^ The public is inundated with inaccurate and imprecise information about brain death in lay media, which sometimes portrays brain death as neurological impairment rather than death.^141^ This renders clinicians’ job of explaining the clinical and legal standards in plain terms particularly important. As a solution, Chen and LaBuzetta recommend education and protocolized responses that address common questions such as whether DNC is reversible, what causes it, mimicking conditions, steps to the DNC exam, and what follows a declaration of DNC.^142^ Additionally, clinicians may consider allowing families to witness the DNC exam process for additional transparency and closure. This may assist the care team to communicate the complex concept of brain death to patient families more effectively, possibly decreasing the prevalence of cases in which families object to the diagnosis.

## Recommendations

Physicians may be declaring patients dead who are not through either (1) false positives resulting from insufficient clinical testing, or (2) inadvertent misdiagnosis by omitting or misinterpreting key tests. This article suggests that taking steps to address both issues can decrease conflict while preserving the current legal standard for brain death. This section outlines recommendations that will reduce potential errors, strengthen the ethical practice of medicine, and offer practical solutions.

### Maintaining DNC and the Importance of a Legal Bright Line

Neurologists recognize that some patients who are declared DNC are not fully dead, but rather have permanently lost *nearly all* neurological functioning or are *almost dead.* ^143^ Unless states revise their laws, a declaration of death still legally requires that physician to use accepted medical standards to determine the patient has suffered irreversible cessation of *all* functions of the *entire* brain. While clinicians must follow the legal standard, the law should also permit latitude for physician expertise when determining which clinical exams work best to assess the patient’s status whether the patient meets the legal criteria. Sulmasy et al. clarify that clinicians should consider the determination as a *functional* rather than *anatomical* assessment.^144^ The distinction means that clinicians do not need to declare every neuron must be dead, but rather that the patient has irrevocably lost all function of the brain that is critical as a “self-integrating whole.”^145^ Clinical standards are designed to complement – but not unilaterally revise — the current legal definition for death.

Retaining the current legal standard provides a bright line category to understand who is dead or alive, which is important for understanding legal rights and interests. Physicians should provide families the accurate clinical status of patients without distorting the definition of death.^146^ For patients who have suffered a devastating irreversible neurological injury and are close to death, many surrogates may choose to withdraw supportive measures, which does not require a determination of death.^147^ True objections to withdrawing support from patients who are truly, legally DNC appear rare.^148^ Truog suggests that physicians can often overcome family hesitation for patients declared DNC by legal standards by compassionately addressing concerns and guiding supportive conversations to assist families with processing loss and grief.^149^

### Legal Implications of Misdiagnosis

Allegations of misdiagnosis can create potential liability for physicians and the institution.^150^ Liability may include claims such as premature diagnosis of DNC, premature disconnection of support, infliction of emotional distress, battery, and restraining orders to halt the process.^151^ Institutions also have a duty to adequately train physicians, oversee physician practice, and formulate policies and procedures to ensure quality patient care.^152^ Institutions that do not provide adequate training and oversight for physicians that perform DNC exams, or lack institutional policies on guidance for physicians to adhere to legal and accepted medical standards may also face potential corporate negligence claims.

Physicians and institutions should take steps to mitigate the potential for misdiagnosis. Clinical tests described by AAN guidelines are necessary but not sufficient, and several AAN declarations conflict with legal requirements. Physicians who follow the guidelines to declare patients dead who retain neurological functioning may incur potential liability. Reducing the risk of liability may require physicians and institutions to implement additional clinical testing to minimize false positives. Institutions can also create training, policies, and procedures to reduce inadvertent misdiagnosis.

### The Ethical Importance of an Accurate Diagnosis

An accurate diagnosis is not only legally important but upholds fundamental ethical principles. As trusted fiduciaries, physicians have a duty to maintain ethical values and improve the practice of medicine when current practices harm patient interests.

Integrity and Honesty. Clinical standards that pronounce physicians may declare patients DNC despite neuroendocrine functioning perpetuate biologically inaccurate information and denies the plain meaning of key words. The legal standard requires *irreversible* cessation of *all functioning* of the *entire brain* and does not permit exceptions. As DeCock et al. aptly point out, trying to change the definition of death from a biological concept by a consensus statement raises troubling concerns for maintaining objective truth in society.^153^

Trust. This strategy risks undermining the foundation of trust and transparency between physicians, patients, and family members.^154^ Families should be able to rely on physicians to provide straightforward, clear and accurate information about the DNC exam and declaration of death. This includes acknowledging that clinical understanding of brain death has evolved and improved. Families rely on physician competence to communicate what they know and admit uncertainties. Here, reducing uncertainty requires considering and implementing additional diagnostic standards to increase accuracy, rather than remaining constrained to current practices.

Nonmaleficence. Moving the goalpost to include patients who are almost dead within the legal definition of dead will likely compound and amplify existing errors of misdiagnosis, which harms patients. Declaring patients dead who are not in fact dead violates the very essence of intrinsic human dignity — the person is no longer a *person* close to death, but a *thing* (e.g. corpse to process and move, or an organ to retrieve).^155^ In instances where the patient is an organ donor, this entails the ultimate violation of nonmaleficence: if the patient is declared DNC but retains neurological functioning, the patient dies from the process of organ retrieval.

Moral Certainty. Death impacts not only the patient but involves the larger context of the patient’s family. Families naturally struggle processing loss and grief. Increasing the accuracy of DNC exams will permit physicians to state to families with confidence they are certain that the patient’s neurological damage is irreversible, total, the patient is in fact dead, and no other interventions would have changed this outcome.

Moral Distress. Clinical standards should not prompt physicians to document, announce, or communicate biologically or legally inaccurate information. Sulmasy et al. highlight in cases where patients retain hypothalamic functioning, this would require physicians to tell families they do not understand what they see plainly with their own eyes.^156^ This constrains how the physician communicates with the family and would require physicians to affirm a position that contradicts with biological reality.

### Practical Solutions to Reduce False Positives and Inadvertent Misdiagnosis

Institutions can take proactive measures to reduce both insufficient testing and inadvertent misdiagnosis from omitting steps or misinterpreting tests. First, institutions should consider how to enhance DNC exams, such as by adding assessment of hypothalamic functioning in institutional policy. Second, standardized institutional policies should integrate the legally compliant components of current clinical guidelines to address inadvertent misdiagnosis. For example, this could include creating protocols to ensure physicians screen out mimicking conditions and provide instructions to indicate that breathing during an apnea test means the patient is alive and cannot be declared DNC.

Health administration teams can help bridge the gap between current testing methods and best practices. Health administration serves a vital role both in anticipating risks and mitigating potential harm by employing procedures and policies to ensure compliance with legal requirements while improving clinical care. Institutions can implement multidisciplinary solutions in conjunction with medical education, legal departments and technology teams.

One suggestion for reducing unintentional errors is for health administrators to establish uniform training or credentialing programs for physicians eligible to declare DNC.^157^ This would ensure they are aware of legal requirements, current guidelines, and how to resolve discrepancies where clinical guidelines conflict with the legal requirements.

Braksick et al. found 23.9% of physicians surveyed who declare DNC reported no formal training, while Biel and Durrant reported training in only 27% of residents.^158^ Though further education and training are important, the question remains what type of training would be most effective. Bernat and Brust stipulate that lectures and videos are helpful yet ultimately insufficient; however, several authors have demonstrated the potential for didactic learning and the use of simulation with role playing.^159^ Simulation instruction should include how the exam should be conducted, how findings should be recorded, as well as how to discuss information with grieving family members.^160^ Simulations of these family member interactions can include elaborating on patient condition, addressing family concerns and emotions, and responding to follow up questions.^161^

A formal credentialing process is another possible strategy for standardizing practice, where health administration teams can implement required training for physicians to perform DNC assessment and diagnosis.^162^ This credentialing process must follow appropriate state laws that specify requirements for certain types of providers, such as a neurologist or neurosurgeon, to assess DNC.

Finally, checklists serve as an additional tool in addressing inadvertent error or missed steps. Health administrators can work with medical education, the legal department, and technology teams to develop a checklist that can be integrated into the electronic health record. The Neurocritical Care Society Task Force provides sample checklists that could be used to inform this process as a component of its Brain Death Toolkit.^163^ Checklists should include accounting for common errors described in recent literature and will provide consistency in practice.^164^ Different checklists should be used for adult and pediatric patient populations.^165^ Checklists should also be updated as guidelines are revised. Checklists should include, but should not be limited to, identifying the etiology or cause of recent brain injury, documenting persistent nonfunction, considering and ruling out confounding factors, steps for clinical testing and documentation, apnea testing, ancillary testing only where indicated or appropriate, and documentation of questions and communication with family members.^166^ Checklists can assist with compliance and documentation as well as reduce omissions that lead to inadvertent misdiagnosis.

## Conclusion

Public policy requires a bright line to determine when patients are alive or dead in clinical practice. Despite clear legal standards set forth in the UDDA, state implementation of this model reflects variations, ethical tensions, and unresolved questions. Part of the controversy acknowledges that current clinical exams do not accurately measure for irreversible loss of all neurological functioning. The solution, then, is that physicians and institutions must improve DNC exams to reduce both false positives and inadvertent misdiagnosis. Multiple stakeholders and the Uniform Law Commission have offered a variety of policy suggestions to modify the UDDA or state laws, but have not reached consensus to change the law. Despite AAN’s clinical guidelines, physicians and institutions should note the law generally still requires: (1) informed consent for patient interventions and evaluations in most instances (or possibly informed dissent); and (2) physicians must determine whether the patient has suffered irreversible cessation of *all functions* of the *entire brain*, which precludes the exceptions stated by AAN. Maintaining a clear, consistent, and reliable standard for DNC will reduce potential error and conflict arising from misdiagnosis and enhance the ethical practice of medicine.
